# Education and household income and carotid intima-media thickness in Japan: baseline data from the Aidai Cohort Study in Yawatahama, Uchiko, Seiyo, and Ainan

**DOI:** 10.1186/s12199-021-01011-6

**Published:** 2021-09-09

**Authors:** Yoshihiro Miyake, Keiko Tanaka, Hidenori Senba, Yasuko Hasebe, Toyohisa Miyata, Takashi Higaki, Eizen Kimura, Bunzo Matsuura, Ryuichi Kawamoto

**Affiliations:** 1 Department of Epidemiology and Preventive Medicine, Ehime University Graduate School of Medicine, Toon, Japan; 2 Research Promotion Unit, Translation Research Center, Ehime University Hospital, Toon, Japan; 3 Center for Data Science, Ehime University, Matsuyama, Japan; 4 Department of Internal Medicine, Matsuyama Shimin Hospital, Matsuyama, Japan; 5 Junpu Health Care Center, Matsuyama, Japan; 6 Department of Regional Pediatrics and Perinatology, Ehime University Graduate School of Medicine, Toon, Japan; 7 Department of Medical Informatics, Ehime University Graduate School of Medicine, Toon, Japan; 8 Department of Lifestyle-Related Medicine and Endocrinology, Ehime University Graduate School of Medicine, Toon, Japan; 9 Department of Community Medicine, Ehime University Graduate School of Medicine, Toon, Japan

**Keywords:** Carotid intima-media thickness, Cross-sectional study, Education, Household income, Japanese

## Abstract

**Background:**

Epidemiological evidence for the relationship between education and income and carotid intima-media thickness (CIMT) has been limited and inconsistent. The present cross-sectional study investigated this issue using baseline data from the Aidai Cohort Study.

**Methods:**

Study subjects were 2012 Japanese men and women aged 34−88 years. Right and left CIMT were measured at the common carotid artery using an automated carotid ultrasonography device. Maximum CIMT was defined as the largest CIMT value in either the left or right common carotid artery. Carotid wall thickening was defined as a maximum CIMT value > 1.0 mm.

**Results:**

The prevalence of carotid wall thickening was 13.0%. In participants under 60 years of age (*n* = 703) and in those aged 60 to 69 years (*n* = 837), neither education nor household income was associated with carotid wall thickening or with maximum CIMT. Among those aged 70 years or older (*n* = 472), however, higher educational level, but not household income, was independently related to a lower prevalence of carotid wall thickening: the multivariate-adjusted odds ratio for high vs. low educational level was 0.43 (95% confidence interval 0.21−0.83, *p* for trend = 0.01). A significant inverse association was observed between education, but not household income, and maximum CIMT (*p* for trend = 0.006).

**Conclusions:**

Higher educational level may be associated with a lower prevalence of carotid wall thickening and a decrease in maximum CIMT only in participants aged 70 years or older.

**Supplementary Information:**

The online version contains supplementary material available at 10.1186/s12199-021-01011-6.

## Introduction

Ischemic heart disease and stroke are the first and second leading causes of death, responsible for approximately 16% and 11%, respectively, of the world’s total deaths [1]. The main underlying cause of cardiovascular disease (CVD) is atherosclerosis, and imaging protocols for subclinical atherosclerosis such as carotid ultrasound measurements of carotid intima-media thickness (CIMT) may improve our ability to assess an individual’s risk of future CVD [2]. Additionally, however, low socioeconomic status has been related to the risk of CVD and may increase the cardiovascular risk to a degree comparable to traditional risk factors, yet the contribution of socioeconomic status to CVD risk is not completely understood [3].

Several epidemiological studies have investigated the relationship between socioeconomic status, specifically, education and income, and CIMT, but their results have been inconsistent [4–12], and epidemiological evidence on this issue is lacking in Japan. To address this, the present cross-sectional study investigated the association of education and household income with carotid wall thickening and maximum CIMT in middle-aged and older Japanese individuals using baseline data from the Aidai Cohort Study (AICOS) conducted in Yawatahama, Uchiko, Seiyo, and Ainan. Since the average education level and household income have increased dramatically over the last half century in Japan, such associations were also assessed in each of three age groups (< 60, 60−69, and ≥ 70 years) separately.

## Methods

### Study population

The AICOS is an ongoing population-based prospective study [13–15]. The baseline survey of the AICOS was commenced in 2015 and is still being completed by new participants as they enroll. The present cross-sectional study was conducted using data obtained in Yawatahama City in 2015, Uchiko Town in 2016, and Seiyo City and Ainan Town in 2017. Yawatahama City, Uchiko Town, Seiyo City, and Ainan Town, with total populations of nearly 36,000; 17,000; 38,000, and 22,000, respectively, are four of the twenty municipalities in Ehime Prefecture on Shikoku Island, which is located south of Japan’s Main Island. In Yawatahama City in 2015, Uchiko Town in 2016, Seiyo City in 2017, and Ainan Town in 2017, 798, 347, 524, and 755 study subjects, respectively, were recruited from among those who had received health checkups conducted by their city of residence or through one of several alternative recruiting procedures. Ultimately, 2424 participants aged 33−89 years (894 men aged 35−89 years and 1530 women aged 33−85 years) gave written informed consent and completed the baseline questionnaire. Excluded were 270 participants because of missing or incomplete data on the factors under study. Another 142 study subjects with a self-reported history of CVD were also excluded. The final sample for analysis consisted of 2012 participants (724 men aged 35−88 years and 1288 women aged 34−85 years). The AICOS was approved by the ethics committee of the Ehime University Graduate School of Medicine.

### Measurements

Using an automated onscreen carotid ultrasonography device (GM-72P00A [The CardioHealth Station]; Panasonic Healthcare Co., Ltd., Ehime, Japan), right and left CIMT were measured at the common carotid artery by trained laboratory technicians or medical doctors within a limited timeframe. The UK Biobank study has demonstrated the excellent reproducibility and face validity of this automated device [16]. Each participant’s maximum CIMT was defined as the largest CIMT value in either the left or the right common carotid artery. Carotid wall thickening was defined as a maximum CIMT > 1.0 mm in keeping with the guidelines proposed by the Japan Academy of Neurosonology and the Japan Society of Ultrasonics in Medicine [17, 18].

A self-administered questionnaire elicited information on age; sex; smoking habits; alcohol drinking habits; physical activity; current use of antihypertensive, cholesterol-lowering, and diabetic medications; employment; education; and household income. Leisure time physical activity was defined as present if the study subjects had engaged in at least 30 min of any type of moderate-to-vigorous physical activity such as brisk walking, playing golf, gardening, jogging, or playing tennis at least once a week. An automated sphygmomanometer was used to take two blood pressure measurements, each with the study subject in the sitting position after at least 5 min of rest; the second measurement was used for the current study. Hypertension was defined as systolic blood pressure ≥ 140 mmHg, diastolic blood pressure ≥ 90 mmHg, or current use of antihypertensive medication. Blood samples were collected from an antecubital vein after an overnight fast. Serum low-density cholesterol, high-density lipoprotein cholesterol, triglyceride concentrations, plasma glucose concentrations, and hemoglobin A1c levels were measured at an external laboratory (Shikoku Chuken, Ehime, Japan). Dyslipidemia was defined as a serum low-density lipoprotein cholesterol concentration ≥ 140 mg/dL, high-density lipoprotein cholesterol concentration < 40 mg/dL, triglyceride concentration ≥ 150 mg/dL, or current use of cholesterol-lowering medication. Diabetes mellitus was defined as a fasting plasma glucose level ≥ 126 mg/dL, hemoglobin A1c level ≥ 6.5%, or current use of diabetic medication. Body height and weight were measured in light clothing without shoes, and body mass index (BMI) was calculated as body weight in kilograms divided by height in meters squared. Waist circumference was measured at the umbilical level with the participant in a standing position.

### Statistical analysis

Age, sex, smoking status, alcohol consumption, leisure time physical activity, hypertension, dyslipidemia, diabetes mellitus, BMI, waist circumference, and employment were selected a priori as potential confounding factors. Age, BMI, and waist circumference were used as continuous variables. Multiple logistic regression analysis was used to estimate adjusted odds ratios (ORs) and 95% confidence intervals (CIs) of carotid wall thickening according to educational level and household income. Analysis of covariance was used to calculate adjusted means of the maximum CIMT according to educational level and household income with allowance for confounding factors. Trend of an association was assessed by a multiple logistic regression model or multiple linear regression analysis assigning consecutive integers to the categories of the exposure variables. *p* values (two-sided) less than 0.05 were regarded as statistically significant. Since the distribution of the maximum CIMT was skewed to the right side, natural logarithms of the values were used so that the presented mean maximum CIMT values and their 95% CIs are geometric. All computations were performed using SAS software version 9.4 (SAS Institute, Inc., Cary, NC, USA).

## Results

Among the 2012 study subjects, the maximum CIMT were distributed over a wide range (0.423−1.925 mm). The median and 95th percentile values were 0.770 and 1.116 mm, respectively. The geometric mean of the maximum CIMT for the population was 0.783 (95% CI 0.776−0.790) mm. The prevalence of carotid wall thickening (the maximum CIMT > 1.0 mm) was 13.0% (Table 1). The median age was 64.0 years and the proportion of men was 36.0%. Educational level was inversely related to age, hypertension, diabetes mellitus, body mass index, and waist circumference and positively associated with current alcohol consumption and employment. Characteristics of the 2012 study subjects in relation to educational level by age group are shown in Supplementary Table 1.

**Table 1:** Characteristics of 2012 study subjects in relation to educational level^a^

Variables	Total (*n* = 2012)	Education^b^			*p* for trend^c^
		Low (*n* = 286)	Medium (*n* = 946)	High (*n* = 780)	
Age, years	64.0 (55.0−69.0)	70.0 (66.0−73.0)	65.0 (58.0−69.0)	59.0 (50.0−66.0)	< 0.0001
Male gender	724 (36.0)	101 (35.3)	344 (36.4)	279 (35.8)	0.99
Smoking status					0.52
Never	1381 (68.6)	200 (69.9)	650 (68.7)	531 (68.1)	
Former	489 (24.3)	66 (23.1)	233 (24.6)	190 (24.4)	
Current	142 (7.1)	20 (7.0)	63 (6.7)	59 (7.6)	
Alcohol consumption					< 0.0001
Never	864 (42.9)	154 (53.9)	415 (43.9)	295 (37.8)	
Former	127 (6.3)	16 (5.6)	66 (7.0)	45 (5.8)	
Current	1021 (50.8)	116 (40.6)	465 (49.2)	440 (56.4)	
Leisure time physical activity	886 (44.0)	136 (47.6)	420 (44.4)	330 (42.3)	0.12
Hypertension	850 (42.3)	164 (57.3)	435 (46.0)	251 (32.2)	< 0.0001
Dyslipidemia	1049 (52.1)	155 (54.2)	509 (53.8)	385 (49.4)	0.07
Diabetes mellitus	157 (7.8)	28 (9.8)	81 (8.6)	48 (6.2)	0.02
Body mass index, kg/m^2^	23.0 (21.0−25.2)	23.7 (21.8−25.8)	22.9 (20.9−25.0)	22.7 (20.8−25.2)	0.002
Waist circumference, cm	82.5 (76.5−89.0)	84.1 (78.5−91.0)	82.5 (76.5−88.4)	82.0 (75.7−88.6)	0.0001
Employment	1165 (57.9)	110 (38.5)	512 (54.1)	543 (69.6)	< 0.0001
Maximum carotid intima-media thickness, mm	0.770 (0.693−0.885)	0.847 (0.731−0.962)	0.785 (0.693−0.885)	0.731 (0.654−0.847)	< 0.0001
Carotid wall thickening	261 (13.0)	59 (20.6)	130 (13.7)	72 (9.2)	< 0.0001

^a^Values are medians (interquartile ranges) for continuous variables and numbers (percentages) of subjects for categorical variables

^b^Low: junior high school; medium: high school; high: junior college, vocational technical school or university

^c^For continuous variables, a linear trend test was used; for categorical variables, a Mantel-Haenszel *χ*^2^-test was used

Table 2 gives ORs and 95% CIs for carotid wall thickening and adjusted geometric means of the maximum CIMT in relation to education and household income. Among the 2012 participants, education was not related to the prevalence of carotid wall thickening in the multivariate model. Between education and maximum CIMT, a significant inverse association appeared after adjustment for age and sex, but this association fell just short of the significance level after multivariate adjustment (*p* for trend = 0.07). No material relationships were observed between household income and carotid wall thickening or maximum CIMT in the multivariate model. No significant multiplicative interactions were found between sex and education or household income with regard to carotid wall thickening (*p* for interaction = 0.13 and 0.17, respectively). When study subjects were classified into three age groups (< 60, 60−69, and ≥ 70 years), neither education nor household income was associated with carotid wall thickening or the maximum CIMT among study subjects under 60 years of age or among those aged 60 to 69 years in the multivariate model. Among those aged 70 years or older, on the other hand, high education level was independently related to a lower prevalence of carotid wall thickening compared with low education level: the multivariate-adjusted OR was 0.43 (95% CI 0.21−0.83, *p* for trend = 0.01). The 70-and-over age group also showed a significant inverse association between education and maximum CIMT: among those with low and high educational levels, the multivariate-adjusted geometric means of maximum CIMT were 0.893 and 0.829 mm, respectively (*p* for trend = 0.006). Household income was not related to carotid wall thickening or to maximum CIMT. Multiplicative interaction between education and overall age with respect to carotid wall thickening was of borderline significance (*p* for interaction = 0.06).

**Table 2:** Adjusted ORs and 95% CIs for carotid wall thickening and adjusted geometric means of the maximum carotid intima-media thickness in relation to education and household income

Variable	Prevalence (%)	Age- and sex-adjusted OR (95% CI)	Multivariate-adjusted OR (95% CI)^a^	Age- and sex-adjusted mean of maximum intima-media thickness, mm (95% CI)	Multivariate-adjusted mean of maximum intima-media thickness, mm (95% CI)^a^
**Overall (*n*= 2012)**					
Education^b^					
Low	59/286 (20.6)	1.00	1.00	0.796 (0.778−0.814)	0.790 (0.773−0.809)
Medium	130/946 (13.7)	0.86 (0.60−1.24)	0.90 (0.62−1.30)	0.788 (0.779−0.798)	0.788 (0.778−0.797)
High	72/780 (9.2)	0.76 (0.51−1.14)	0.80 (0.53−1.21)	0.772 (0.761−0.782)	0.774 (0.764−0.785)
*p* for trend		0.18	0.28	0.01	0.07
Household income (Japanese million yen/year)^c^					
< 3	110/687 (16.0)	1.00	1.00	0.786 (0.774−0.797)	0.785 (0.773−0.796)
3−< 5	92/687 (13.4)	0.93 (0.68−1.27)	1.00 (0.73−1.38)	0.784 (0.772−0.795)	0.786 (0.775−0.797)
≥ 5	59/638 (9.3)	0.93 (0.65−1.34)	0.95 (0.64−1.38)	0.779 (0.767−0.791)	0.778 (0.766−0.789)
*p* for trend		0.67	0.80	0.42	0.43
**< 60 years of age (*n*= 703)**					
Education^b^					
Low	1/15 (6.7)	1.00	1.00	0.711 (0.648−0.780)	0.684 (0.625−0.749)
Medium	12/274 (4.4)	0.64 (0.11−12.38)	1.03 (0.14−21.61)	0.718 (0.703−0.734)	0.713 (0.698−0.728)
High	17/414 (4.1)	0.65 (0.11−12.35)	1.22 (0.17−25.36)	0.695 (0.683−0.708)	0.700 (0.688−0.712)
*p* for trend		0.87	0.69	0.03	0.33
Household income (Japanese million yen/year)^c^					
< 3	4/121 (3.3)	1.00	1.00	0.699 (0.676−0.722)	0.696 (0.674−0.719)
3−< 5	8/211 (3.8)	1.06 (0.32−4.10)	1.16 (0.34−4.66)	0.709 (0.691−0.726)	0.710 (0.693−0.727)
≥ 5	18/371 (4.9)	1.45 (0.52−5.18)	1.26 (0.41−4.79)	0.704 (0.691−0.717)	0.704 (0.692−0.717)
*p* for trend		0.41	0.71	0.85	0.71
**60−69 years of age (*n*= 837)**					
Education^b^					
Low	15/127 (11.8)	1.00	1.00	0.801 (0.775−0.828)	0.797 (0.771−0.824)
Medium	62/437 (14.2)	1.33 (0.74−2.55)	1.46 (0.79−2.85)	0.807 (0.793−0.822)	0.808 (0.794−0.822)
High	39/273 (14.3)	1.41 (0.75−2.79)	1.53 (0.79−3.09)	0.809 (0.791−0.827)	0.810 (0.792−0.828)
*p* for trend		0.35	0.27	0.68	0.51
Household income (Japanese million yen/year)^c^					
< 3	50/336 (14.9)	1.00	1.00	0.814 (0.798−0.831)	0.814 (0.798−0.831)
3−< 5	39/297 (13.1)	0.85 (0.54−1.35)	0.93 (0.57−1.51)	0.802 (0.785−0.819)	0.804 (0.787−0.822)
≥ 5	27/204 (13.2)	0.86 (0.51−1.43)	0.91 (0.52−1.57)	0.802 (0.781−0.823)	0.798 (0.778−0.820)
*p* for trend		0.51	0.72	0.32	0.23
**≥ 70 years of age (*n*= 472)**					
Education^b^					
Low	43/144 (29.9)	1.00	1.00	0.893 (0.864−0.923)	0.893 (0.864−0.923)
Medium	56/235 (23.8)	0.73 (0.45−1.16)	0.69 (0.42−1.14)	0.869 (0.847−0.891)	0.868 (0.847−0.891)
High	16/93 (17.2)	0.46 (0.24−0.87)	0.43 (0.21−0.83)	0.829 (0.796−0.863)	0.829 (0.796−0.863)
*p* for trend		0.02	0.01	0.006	0.006
Household income (Japanese million yen/year)^c^					
< 3	56/230 (24.4)	1.00	1.00	0.868 (0.845−0.891)	0.866 (0.844−0.889)
3−< 5	45/179 (25.1)	1.03 (0.65−1.63)	1.08 (0.67−1.74)	0.873 (0.848−0.900)	0.876 (0.851−0.903)
≥ 5	14/63 (22.2)	0.88 (0.44−1.69)	0.89 (0.43−1.74)	0.853 (0.811−0.896)	0.850 (0.809−0.894)
*p* for trend		0.81	0.89	0.72	0.78

*CI* confidence interval, *OR* odds ratio

^a^Adjustment for age, sex, smoking status, alcohol consumption, leisure time physical activity, hypertension, dyslipidemia, diabetes mellitus, body mass index, waist circumference, and employment

^b^Low: junior high school; medium: high school; high: junior college, vocational technical school or university

^c^US $1 = 104 yen

## Discussion

To our knowledge, this is the first study in Japan to investigate the association of education and household income with CIMT. The current cross-sectional study showed that educational level, but not household income, is independently inversely related to both the prevalence of carotid wall thickening and the maximum CIMT in participants aged 70 years or older who are free of self-reported clinical cardiovascular disease. No measurable associations were observed between education or household income and carotid wall thickening or maximum CIMT in all age groups considered together, in participants younger than 60 years, or in those aged 60 to 69 years.

A cross-sectional study of 4524 African Americans (mean age 54 years, 64% female) showed that education, but not income, was significantly inversely associated with CIMT [4]. In a prospective study of 1402 US women aged 42 to 52 years at baseline, no relationships were found between education or income over 12 years and CIMT [5]. In a cross-sectional study of 5474 older French persons (mean age 73 years, 63% female), education was significantly inversely related to CIMT in men but not in women [6]. In a Finnish cohort study, at baseline, there was no association between education and CIMT among 1813 subjects aged 24–39 years, but education was significantly inversely associated with changes in CIMT over 6 years [7]. A cross-sectional study of 2042 US subjects (mean age 68 years, 57% female) showed significant inverse associations of education and income with CIMT [8]. In the Multi-Ethnic Study of Atherosclerosis conducted in the USA, a significant inverse relationship was observed between education and CIMT among White participants (*n* = 2624), but not among Chinese (*n* = 803), Black (*n* = 1895), or Hispanic (*n* = 1492) participants, while there were no associations between income and CIMT regardless of race/ethnicity [9]. A cross-sectional study of 4176 Swedish participants aged 46–68 years (59% female) found that, while education was not related to CIMT in men or women, it was significantly inversely associated with carotid stenosis (reduction of the luminal diameter by 15% or greater) in women but not in men [10]. A significant inverse relationship was found between education, but not income, and CIMT in a cross-sectional study of 1140 Finnish participants [11]. No association was shown between education and CIMT in a cross-sectional study of 3703 subjects from five European countries (median age 64 years, 52% female) each subject having at least three vascular risk factors but free from previous cardiovascular and cerebrovascular events [12]. The current results are in partial agreement with these findings.

The mechanisms underlying the observed inverse association between education and CIMT only among those aged 70 years or older are likely numerous. Compared with income, education level is likely to be the more stable aspect of socioeconomic status because it is typically established at an early age and tends to remain the same over time [19]. This might explain the inverse association between education level and CIMT, especially in those aged 70 years or older, through better health awareness in more educated individuals. As Table 2 shows, the proportion of low educational levels increased with age while the proportion of high educational levels decreased with age; the proportions of participants with low and high education levels were 2.1% and 58.9%, respectively, among those under 60 years of age; 15.2% and 32.6%, respectively, among those aged 60 to 69 years; and 30.5% and 19.7%, respectively, among those aged 70 years or older. The fact that each generation of residents of Japan has found it easier to achieve higher educational levels due to the nation’s economic growth might account for the weakness of the association between educational level and health findings at younger ages. The relative rarity of a high educational level among more aged persons might explain the stronger inverse association in this group, leading to our finding that the inverse association between education and CIMT was significant only among those aged 70 years or older. Alternatively, the observed inverse association might simply be a chance phenomenon.

The current study had methodological advantages in that participants were homogeneous with respect to their residential area, in that we used an automated onscreen carotid ultrasound system, and in that we adjusted for a variety of potential confounding factors.

Some weaknesses of the present study should be borne in mind. The present study design was cross-sectional; thus, our results should not be interpreted as a cause-effect association. Selection bias was unavoidable. The participation rate must have been low; additionally, the participation rate could not be estimated because the exact number of eligible subjects was not available. The present study subjects were probably not representative of the Japanese general population. For example, the educational levels of the present subjects were higher than those of the general population. According to a population census conducted in 2010 in Ehime Prefecture [20], the proportions of persons aged 60−69 years with low, medium, and high educational levels and an unknown educational level were 28.2%, 48.6%, 19.0%, and 4.2%, respectively, in men and 26.7%, 56.4%, 12.9%, and 4.0%, respectively, in women. The corresponding figures in the present study for persons aged 60−69 years were 13.2%, 52.7%, 34.1%, and 0.0%, respectively, in men and 16.3%, 51.9%, 31.8%, and 0.0%, respectively, in women.

We have no data on the validity of self-reported information on education and household income, but any potential non-differential exposure misclassification would result in a bias towards the null. A potential limitation of the automated onscreen carotid ultrasonography device used in the present study is the lack of quantified plaque present in the images. CIMT and plaque are phenotypically distinct findings that both indicate increased vascular risk, yet CIMT without plaque remains a significant marker of increased risk of vascular events and significantly predicts plaque occurrence [21]. The lack of a significant inverse relationship between education and carotid wall thickening or maximum CIMT among the overall study subjects might be ascribed to insufficient statistical power. Residual confounding effects could not be overcome although several confounding factors were controlled for.

## Conclusion

The present cross-sectional study in Japan suggests that higher educational levels may be associated with a lower prevalence of carotid wall thickening and a smaller maximum CIMT only in participants aged 70 years or older, whereas household income was not related to these outcomes regardless of age. Further well-designed investigations of the effects of education and household income on CIMT are required, especially in Asian populations.

## Supplementary Information

**Additional file 1.** Supplementary Table 1. Characteristics of the 2012 study subjects in relation to educational level by age group^a^.

## Data Availability

The data sets generated and analyzed in the present study are not publicly available because we did not obtain informed consent from the participants for the open use of individual data.

## Abbreviations

AICOS: Aidai Cohort Study
BMI: Body mass index
CVD: Cardiovascular disease
CI: Confidence interval
CIMT: Carotid intima-media thickness
OR: Odds ratio
