# Supplementary material for: Education and household income and carotid intima-media thickness in Japan: baseline data from the Aidai Cohort Study in Yawatahama, Uchiko, Seiyo, and Ainan
Source: Environ Health Prev Med. 2021 Sep 9;26:88. doi: 10.1186/s12199-021-01011-6 (PMC8428125; doi:10.1186/s12199-021-01011-6)
Supplement: Supplementary file 1 — Additional file 1. Supplementary Table 1. Characteristics of the 2012 study subjects in relation to educational level by age groupa. [file 12199_2021_1011_MOESM1_ESM.docx]

Supplementary Table 1. Characteristics of the 2012 study subjects in relation to educational level by age group^a^

|  | Total | Education^b^ |  |  |  |
| --- | --- | --- | --- | --- | --- |
| Variables |  | Low | Medium | High | *p* for trend^c^ |
| **< 60 years of age** | **(n = 703)** | **(n = 15)** | **(n = 274)** | **(n = 414)** |  |
| Age, years | 51.0 (45.0−56.0) | 53.0 (44.0−59.0) | 52.0 (46.0−56.0) | 51.0 (45.0−56.0) | 0.054 |
| Male gender | 225 (32.0) | 5 (33.3) | 89 (32.5) | 131 (31.6) | 0.80 |
| Smoking status |  |  |  |  | 0.28 |
| Never | 481 (68.4) | 7 (46.7) | 186 (67.9) | 288 (69.6) |  |
| Former | 149 (21.2) | 6 (40.0) | 58 (21.2) | 85 (20.5) |  |
| Current | 73 (10.4) | 2 (13.3) | 30 (11.0) | 41 (9.9) |  |
| Alcohol consumption |  |  |  |  | 0.006 |
| Never | 231 (32.9) | 8 (53.3) | 101 (36.9) | 122 (29.5) |  |
| Former | 41 (5.8) | 1 (6.7) | 18 (6.6) | 22 (5.3) |  |
| Current | 431 (61.3) | 6 (40.0) | 155 (56.6) | 270 (65.2) |  |
| Leisure time physical activity | 192 (27.3) | 0 (0.0) | 76 (27.7) | 116 (28.0) | 0.27 |
| Hypertension | 176 (25.0) | 8 (53.3) | 85 (31.0) | 83 (20.1) | < 0.0001 |
| Dyslipidemia | 312 (44.4) | 6 (40.0) | 128 (46.7) | 178 (43.0) | 0.47 |
| Diabetes mellitus | 25 (3.6) | 1 (6.7) | 11 (4.0) | 13 (3.1) | 0.41 |
| Body mass index, kg/m^2^ | 22.8 (20.7−25.6) | 24.0 (21.8−31.3) | 23.3 (21.1−26.0) | 22.5 (20.5−25.2) | 0.002 |
| Waist circumference, cm | 81.0 (74.8−88.5) | 82.5 (77.0−101.2) | 81.8 (75.0−88.8) | 80.6 (74.3−87.5) | 0.01 |
| Employment | 625 (88.9) | 11 (73.3) | 239 (87.2) | 375 (90.6) | 0.04 |
| Maximum carotid intima-media thickness, mm | 0.693 (0.616−0.808) | 0.693 (0.577−0.847) | 0.731 (0.616−0.808) | 0.693 (0.616−0.808) | 0.008 |
| Carotid wall thickening | 30 (4.3) | 1 (6.7) | 12 (4.4) | 17 (4.1) | 0.72 |
| **60−69 years of age** | **(n = 837)** | **(n = 127)** | **(n = 437)** | **(n = 273)** |  |
| Age, years | 65.0 (63.0−67.0) | 66.0 (65.0−68.0) | 65.0 (63.0−67.0) | 65.0 (62.0−67.0) | < 0.0001 |
| Male gender | 296 (35.4) | 39 (30.7) | 156 (35.7) | 101 (37.0) | 0.26 |
| Smoking status |  |  |  |  | 0.86 |
| Never | 570 (68.1) | 85 (66.9) | 301 (68.9) | 184 (67.4) |  |
| Former | 213 (25.5) | 33 (26.0) | 107 (24.5) | 73 (26.7) |  |
| Current | 54 (6.5) | 9 (7.1) | 29 (6.6) | 16 (5.9) |  |
| Alcohol consumption |  |  |  |  | 0.32 |
| Never | 389 (46.5) | 69 (54.3) | 193 (44.2) | 127 (46.5) |  |
| Former | 57 (6.8) | 9 (7.1) | 28 (6.4) | 20 (7.3) |  |
| Current | 391 (46.7) | 49 (38.6) | 216 (49.4) | 126 (46.2) |  |
| Leisure time physical activity | 427 (51.0) | 57 (44.9) | 214 (49.0) | 156 (57.1) | 0.01 |
| Hypertension | 389 (46.5) | 64 (50.4) | 206 (47.1) | 119 (43.6) | 0.18 |
| Dyslipidemia | 489 (58.4) | 77 (60.6) | 252 (57.7) | 160 (58.6) | 0.81 |
| Diabetes mellitus | 68 (8.1) | 8 (6.3) | 40 (9.2) | 20 (7.3) | 0.98 |
| Body mass index, kg/m^2^ | 22.9 (21.0−25.0) | 23.7 (21.9−25.9) | 22.7 (20.8−24.7) | 22.9 (20.9−25.3) | 0.09 |
| Waist circumference, cm | 82.0 (76.8−88.5) | 83.5 (77.8−89.8) | 81.6 (76.4−87.6) | 82.5 (77.0−89.3) | 0.21 |
| Employment | 397 (47.4) | 58 (45.7) | 202 (46.2) | 137 (50.2) | 0.31 |
| Maximum carotid intima-media thickness, mm | 0.808 (0.693−0.924) | 0.808 (0.693−0.924) | 0.808 (0.693−0.924) | 0.808 (0.693−0.924) | 0.94 |
| Carotid wall thickening | 116 (13.9) | 15 (11.8) | 62 (14.2) | 39 (14.3) | 0.57 |
| **≥ 70 years of age** | **(n = 472)** | **(n = 144)** | **(n = 235)** | **(n = 93)** |  |
| Age, years | 73.0 (71.0−76.0) | 73.0 (71.5−76.0) | 73.0 (71.0−75.0) | 73.0 (71.0−76.0) | 0.92 |
| Male gender | 203 (43.0) | 57 (39.6) | 99 (42.1) | 47 (50.5) | 0.11 |
| Smoking status |  |  |  |  | 0.33 |
| Never | 330 (69.9) | 108 (75.0) | 163 (69.4) | 59 (63.4) |  |
| Former | 127 (26.9) | 27 (18.8) | 68 (28.9) | 32 (34.4) |  |
| Current | 15 (3.2) | 9 (6.3) | 4 (1.7) | 2 (2.2) |  |
| Alcohol consumption |  |  |  |  | 0.53 |
| Never | 244 (51.7) | 77 (53.5) | 121 (51.5) | 46 (49.5) |  |
| Former | 29 (6.1) | 6 (4.2) | 20 (8.5) | 3 (3.2) |  |
| Current | 199 (42.2) | 61 (42.4) | 94 (40.0) | 44 (47.3) |  |
| Leisure time physical activity | 267 (56.6) | 79 (54.9) | 130 (55.3) | 58 (62.4) | 0.30 |
| Hypertension | 285 (60.4) | 92 (63.9) | 144 (61.3) | 49 (52.7) | 0.10 |
| Dyslipidemia | 248 (52.5) | 72 (50.0) | 129 (54.9) | 47 (50.5) | 0.81 |
| Diabetes mellitus | 64 (13.6) | 19 (13.2) | 30 (12.8) | 15 (16.1) | 0.58 |
| Body mass index, kg/m^2^ | 23.3 (21.2−25.1) | 23.8 (21.3−25.7) | 23.0 (21.1−24.7) | 23.3 (21.8−25.0) | 0.17 |
| Waist circumference, cm | 84.1 (79.0−89.8) | 85.5 (79.5−91.9) | 84.0 (79.0−88.3) | 84.5 (79.0−91.0) | 0.67 |
| Employment | 143 (30.3) | 41 (28.5) | 71 (30.2) | 31 (33.3) | 0.44 |
| Maximum carotid intima-media thickness, mm | 0.847 (0.770−0.962) | 0.885 (0.770−1.020) | 0.847 (0.770−0.962) | 0.808 (0.731−0.924) | 0.005 |
| Carotid wall thickening | 115 (24.4) | 43 (29.9) | 56 (23.8) | 16 (17.2) | 0.03 |

^a^ Values are medians (interquartile ranges) for continuous variables and numbers (percentages) of subjects for categorical variables.

^b^ Low: junior high school; medium: high school; high: junior college, vocational technical school or university.

^c^ For continuous variables, a linear trend test was used; for categorical variables, a Mantel-Haenszel χ^2^-test was used.
